# Disease progression of cancer patients during COVID-19 pandemic: a comprehensive analytical strategy by time-dependent modelling

**DOI:** 10.1186/s12874-020-01090-z

**Published:** 2020-08-12

**Authors:** Atanu Bhattacharjee, Gajendra K. Vishwakarma, Souvik Banerjee, Sharvari Shukla

**Affiliations:** 1 Section of Biostatistics, Centre for Cancer Epidemiology, Tata Memorial Centre, Mumbai, India; 2 Homi Bhabha National Institute, Mumbai, India; 3 Department of Mathematics & Computing, Indian Institute of Technology (ISM), 826004, Dhanbad, India; 4 Symbiosis Statistical Institute, Symbiosis International University, Pune, India

**Keywords:** COVID-19, Accelerated failure time, Proportional Hazard model, Bayesian, Auto-regression

## Abstract

**Background:**

As the whole world is experiencing the cascading effect of a new pandemic, almost every aspect of modern life has been disrupted. Because of health emergencies during this period, widespread fear has resulted in compromised patient safety, especially for patients with cancer. It is very challenging to treat such cancer patients because of the complexity of providing care and treatment, along with COVID-19. Hence, an effective treatment comparison strategy is needed. We need to have a handy tool to understand cancer progression in this unprecedented scenario. Linking different events of cancer progression is the need of the hour. It is a huge challenge for the development of new methodology.

**Methods:**

This article explores the time lag effect and makes a statistical inference about the best experimental arm using Accelerated Failure Time (AFT) model and regression methods. The work is presented as the occurrence of other events as a hazard rate after the first event (relapse). The time lag effect between the events is linked and analysed.

**Results:**

The results were presented as a comprehensive analytical strategy by joining all disease progression. An AFT model applied with the transition states, and the dependency structure between the gap times was used by the auto-regression model. The effects of arms were compared using the coefficient of auto-regression and accelerated failure time (AFT) models.

**Conclusions:**

We provide the solutions to overcome the issue with intervals between two consecutive events in motivating head and neck cancer (HNC) data. COVID-19 is not going to leave us soon. We have to conduct several cancer clinical trials in the presence of COVID-19. A comprehensive analytical strategy to analyse cancer clinical trial data during COVID-19 pandemic is presented.

## Background

Cancer patients are more prone to develop COVID-19 because they are immunocompromised [1]. Studies have suggested that cancer patients are more susceptible to Coronavirus, whereas individuals without cancer are immunosuppressed. Though the risk of COVID-19 infection varies individually, cancer patients require continuous care and treatment intervention and potential risk of COVID-19 exposure could be fatal [2]. Studies have shown that COVID-19 has created a great challenge to manage the cancer care delivery system [3].

It is essential to assess the patient’s risk of both COVID-19 and tumour control on a case-by-case basis with the patient. Conventionally, the treatment effect of head and neck cancer (HNC) is explored by multiple events like loco-regional control (LRC), progression-free survival (PFS), and overall survival (OS). These events are analysed separately by Kaplan-Meier [4] and the Cox Proportional Hazard (CPH) models [5]. Currently, it is difficult to isolate the reason for death due to Coronavirus or disease progression among cancer patients [6]. Similarly, all ongoing cancer clinical trials cannot stop due to COVID-19 in the long run, and it is challenging to conduct cancer clinical trials [7] in this present environment. Thus, time lag/intervals between different types of events are essential to explore.

In this manuscript, we focused on exploring the time lag effect and studied the statistical inference about the best experimental arm using Accelerated Failure Time (AFT) Model and regression methods. We present our work here for the occurrence of other events as a hazard rate after the first event (relapse). It is known that local relapse biologically triggers cancer progression and death; however, in this study, we have not considered it. As most of the events are likely to be influenced by COVID-19 infection, so it required to establish an integrated analysis.

The relapse triggers disease progression, and further, disease progression accelerates death rate. The study considered two-time points generated as the duration between relapse to progression and duration between progress to death. For these transition periods, we used the CPH and AFT model, which are useful to work on transition states where treatment effect is comparable.

In this study, the statistical model was considered to handle both the previously mentioned time points and explore the relations between gap durations. Further, we applied a CPH model to understand the different types of transition hazard models and the time-varying covariates considered separately. The results presented as a comprehensive analytical strategy. An AFT model applied with the transition states, and we explained the dependency structure between the gap times using auto-regression. The effects of arms compared using the coefficient of auto-regression and AFT models - the complete analysis using Bayesian techniques executed with R open-source software and OpenBUGS.

## Methods

### Dependency modelling

It is difficult to reduce risk and prevent the spread of the COVID-19 virus among vulnerable cancer patients. At the same time, we have to provide treatment to all these several thousand vulnerable cancer patients. Thus, this becomes very challenging to treat patients separately from patients only with COVID-19. There is a very minimal chance that cancer patients will not get infected by COVID-19 in the long run. We have to run several clinical trials in the presence of COVID-19 infection. Disease progression events occurred as loco-regional relapse, progression, and death - the events marked as 1, 2 and 3, respectively. The events ordered, which implies that the loco-regional relapse appeared earlier than progression or death, and death as a terminal event. Here, our interest was to measure the event occurrence rate at each of the interval or gap time between two events. Let *T*_*i*, *j*_ be the actual event time for *i*^th^ individual and *j* denoted different events by 1, 2 or 3. We considered that all the individuals had experienced at least one event. The intervals between two subsequent events were defined as follows:

1 $$ {G}_{i,1}={T}_{i,1}\kern0.5em \mathrm{and}\kern0.5em {G}_{i,\mathrm{j}}=\kern0.5em {T}_{i,\mathrm{j}}-\kern0.5em {T}_{i,\mathrm{j}-1}\kern0.5em \mathrm{for}\kern0.5em i=1,2,\dots \kern0.5em .,n;\kern0.5em j=1,2\kern0.5em \mathrm{and}\kern0.5em {T}_{i,0}\kern0.5em =\kern0.5em 0. $$

In our study, the gap times were assumed to be dependent with ordered events. In order to the dependency structure, we concluded that the 1st event corresponds to *G*_*i*, 1_, the duration from the beginning of the study to the occurrence of the second event, the second event correspond to *G*_*i*, 2_ and so on. So, the dependency structure was presented among *G*_*i*, 1_, *G*_*i*, 2_ etc.

We assumed that a simple linear regression model between *G*_*i*, 1_ and *G*_*i*, 2_. The regression model was.

2 $$ {G}_{i,1}=\kern0.5em {\beta}_{0,0}+\kern0.5em {\beta}_{1,0}{G}_{i,0}\kern0.5em ,\kern0.5em {G}_{i,2}=\kern0.5em {\beta}_{0,1}+\kern0.5em {\beta}_{i,1}{G}_{i,1} $$

We fit two separate linear regressions for two different arms. *β*_1, 0_ and defined the change in *G*_*i*, 2_ for a unit change in *G*_*i*, 1_ for arm 0. The same inference was drawn for *β*_1, 1_ So, ignoring the intercept term in the regression model, the difference between the coefficients *β*_1, 1_- *β*_1, 0_ stated the change in dependent gap time was due to change in the arm. We fit AFT models for *G*_*i*, 1_ and *G*_*i*, 2_ and obtained the corresponding coefficients of the arm to measure the change on events due to variation in treatment.

### AFT model with gap time

The AFT model is a popular alternative of proportional hazard model to analyse survival data [8, 9]. It is also applicable in the current COVID-19 scenario. It is more efficient to model the survival time rather than hazard rate; to observe the dependency pattern between observed times. In the AFT model, it assumed that the effect of the covariate is to accelerate or decelerate the survival duration by some constants. The AFT model is expressed as,

3 $$ {Y}_i=\log\ \left({G}_i\right)=\mu +\beta {x}_i+{\varepsilon}_i. $$

Here, *G*_*i*_ denotes the survival time for *i*^*th*^ individual, *β* is the unknown regression coefficient, *μ* is the intercept term, *x*_*i*_ is the covariate for *i*^*th*^ subject (*i* = 1, 2, …. *n*), *ε*_*i*_ is the error component, *ε*_1_, *ε*_2_, …, *ε*_*n*_ are independent and identically distributed as Normal (0,1). So, given covariates, the response times are independent. In our study, we consider the gap time to fit AFT models for different event occurrences. The gap times (*G*_*i*_) between two consecutive events are model as response variables in eq. (3).

For the AFT model, the survival function is

4 $$ S\left(t|{x}_i\right)={S}_0\Big[\exp \left\{-\left(\mu +\beta {x}_i\right)\right\}t. $$

We considered the Bayesian approach to estimate the parameter estimates for the AFT model obtained from the posterior distributions based on Markov Chain Monte Carlo (MCMC) simulation by Gibbs Sampling method. To conduct data analysis using Bayesian techniques, we need to specify the prior distributions of the parameters. We used independent Gaussian prior distributions with mean 0 and variance 0.001 for the parameter *μ* and other regression coefficient *β*. The models were compared, and the best fit model was decided based on the Akaike Information Criterion (AIC).

The better fit among candidate models performed through the Akaike information criterion (AIC) [10, 11] as

5 $$ AIC=-2\ln \left\{p\left(\hat{\theta}\right)\right\}+2k. $$

The number of parameters is represented by *k*. The random variable and maximum likelihood estimate were presented by *x* and $\hat{\theta}$ where the parameter of interest was defined as *θ*. The minimal value of AIC shows a better fit of the model. The Bayesian extension of the Cox proportional hazard model was presented as

6 $$ P\left(\theta |Y\right)=\frac{P\left(Y|\theta \right)P\left(\theta \right)}{P(Y)}. $$

The term Y was the observed evidence, and the marginal probability of *Y* was defined as *P(Y)*. The prior is *P(θ)* and the likelihood function was *P*(*Y*| *θ*). Mean, standard deviation, credible interval and the highest posterior density (HPD) were computed for each parameter. An alternative of the AIC in the context of Bayesian model selection method was Deviance Information Criteria (DIC) [12, 13]. The Deviance Information Criteria (DIC) was defined as,

7 $$ DIC=-2\ln \left\{p\left(\hat{\theta}\right)\right\}+{p}_D $$

where

8 $$ {p}_D=E\left[-2\ln \left\{p\left(x|\ \hat{\theta}\right)\right\}\right]+2\ln\ \left\{p\left(x|\hat{\theta}\right)\right\}. $$

The DIC estimates the valid number of parameters by the difference of the posterior mean of the deviance and deviance of posterior means.

### Bayesian CPH regression separately for each event

The Cox proportional hazards (Cox PH) model was applied in time-to-event data analysis [14–16]. It was defined as

9 $$ {\lambda}_i\left({Z}_i\right)={\lambda}_0(t)\exp \left({Z}_i\beta \right) $$

or

10 $$ \log\ {\lambda}_i\left({Z}_i\right)=\log\ {\lambda}_0(t)+{Z}_i\beta; i=1,2,\dots, n. $$

For the *i*^th^ patient, the baseline hazard and hazard at time t were defined by *λ*_0_(*t*) and *λ*_*i*_(*t*| *Z*_*i*_), *Z*_*i*_ is the covariate for an *i*^th^ patient with the regression coefficient *β*. The hazard ratio was defined as a predicted hazard function under different predictor variables. The partial likelihood function was adopted to fit the Cox model. A high *p*-value for the coefficient was defined as less significance of the variable of interest. The better fit among candidate models was performed through the Akaike information criterion (AIC) as discussed. Similarly, DIC was used for model comparison while using Bayesian techniques.

We considered different time-to-events in different CPH models with several factors like arm, age, and gender and obtained the posterior means of the parameters through the models provided in Table 1. The CPH was performed as a conventional choice to show time to event data analysis.

**Table 1:** Posterior Estimate generated through different models through Cox PH model

Bayesian Estimate						MLE
Response	Parameter	Posterior Mean (SD)	95% HPD	DIC	PD	
LRC	Arm	−0.31 (0.14)	(−0.61, − 0.03)	2187.7	0.99	− 0.31 (− 0.60, − 0.02)
	Age	−0.20 (0.17)	(− 0.53, 0.13)			
	Gender	0.41 (0.23)	(−0.06, 0.86)			
PFS	Arm	−0.31 (0.13)	(−0.58, − 0.05)	2614.46	0.99	− 0.31 (− 0.57, − 0.04)
	Age	−0.38 (0.15)	(− 0.64, − 0.09)			
	Gender	0.49 (0.22)	(0.05, 0.91)			
OS	Arm	−0.16 (0.13)	(−0.42, 0.08)	2610.64	0.99	−0.16 (− 0.42, 0.08)
	Age	−0.38 (0.15)	(− 0.68, − 0.09)			
	Gender	0.28 (0.20)	(−0.13, 0.66)			

## Results

Dataset was presented to resemble a motivating example of head and neck cancer (HNC). A total of 74 patients treated with two chemotherapeutic arms were illustrated. The clinical trial was aimed to perform the PFS between two types of therapy. The therapies were (I) ‘Arm-A’ (*n* = 43 subjects) or (II) ‘Arm-B’ (*n* = 31 subjects). The covariates considered were (a) Arm, (b) Age and (c) Gender. Subjects were followed continuously, and the occurrence of relapse, disease progression and death were monitored. Data with missing observations were not considered for analysis. The mimic data was uploaded as supplementary file S1.

We considered the duration between treatment initiation to the time of progression or the last follow-up visit for patients who had not progressed – the sequence was defined as RECIST criteria version 1.1. Disease-free survival was considered as the duration while the person experienced complete remission. We found the period between LRC and progression as *T*_1_ and between progression and death as *T*_2_.

One of the aims of the trial was to investigate the best active arm to prolong the PFS. The experiment was continued to explore the loco-regional recurrence and overall survival. In this example, we measured the LRC as the duration between dates of registration to the time of first loco-regional relapse. Similarly, the date of enrolment to date of progression was defined as PFS. The OS was defined as the last date of follow-up or date of death from the date of registration. The CPH hazard model and AFT model were considered for different states in the context of Bayesian frameworks. The states were defined as a dead state (state 3), living with the progressed disease (state 2) and living with loco-regional recurrence, not with distant metastasis/progression (state1). The direct transition from state 1 to state 3 is possible. However, as mentioned earlier, we considered only those patients for which all three states were apparent.

The CPH model applied in this dataset was defined as,

11 $$ \lambda (x)={\lambda}_0(t)\exp \left({\beta}_1\ast \mathrm{Arm}+{\beta}_2\ast \mathrm{Age}+{\beta}_3\ast \mathrm{Gender}\right). $$

**Fig. 1:**
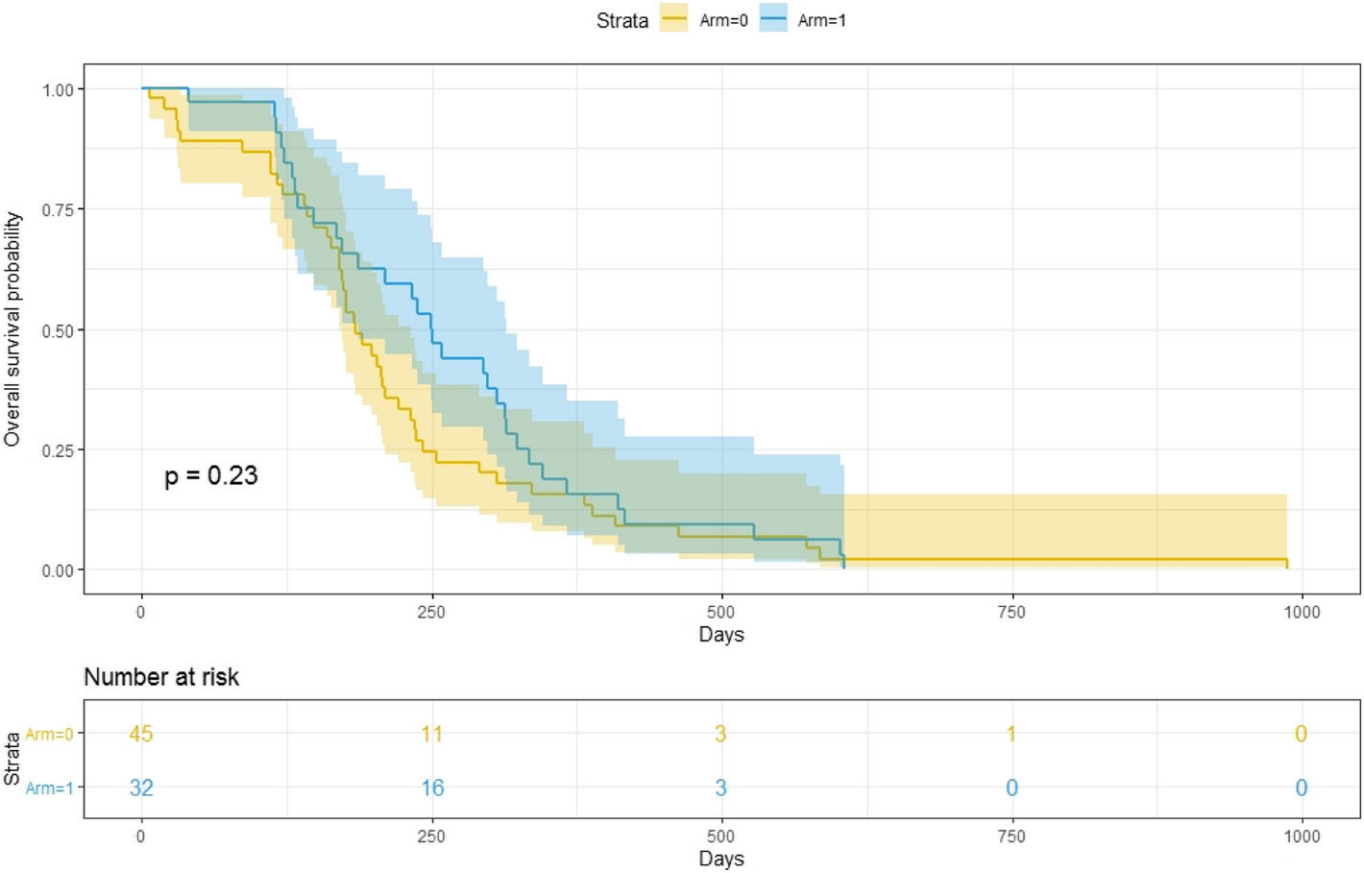
Loco-regional relapse progression

**Fig. 2:**
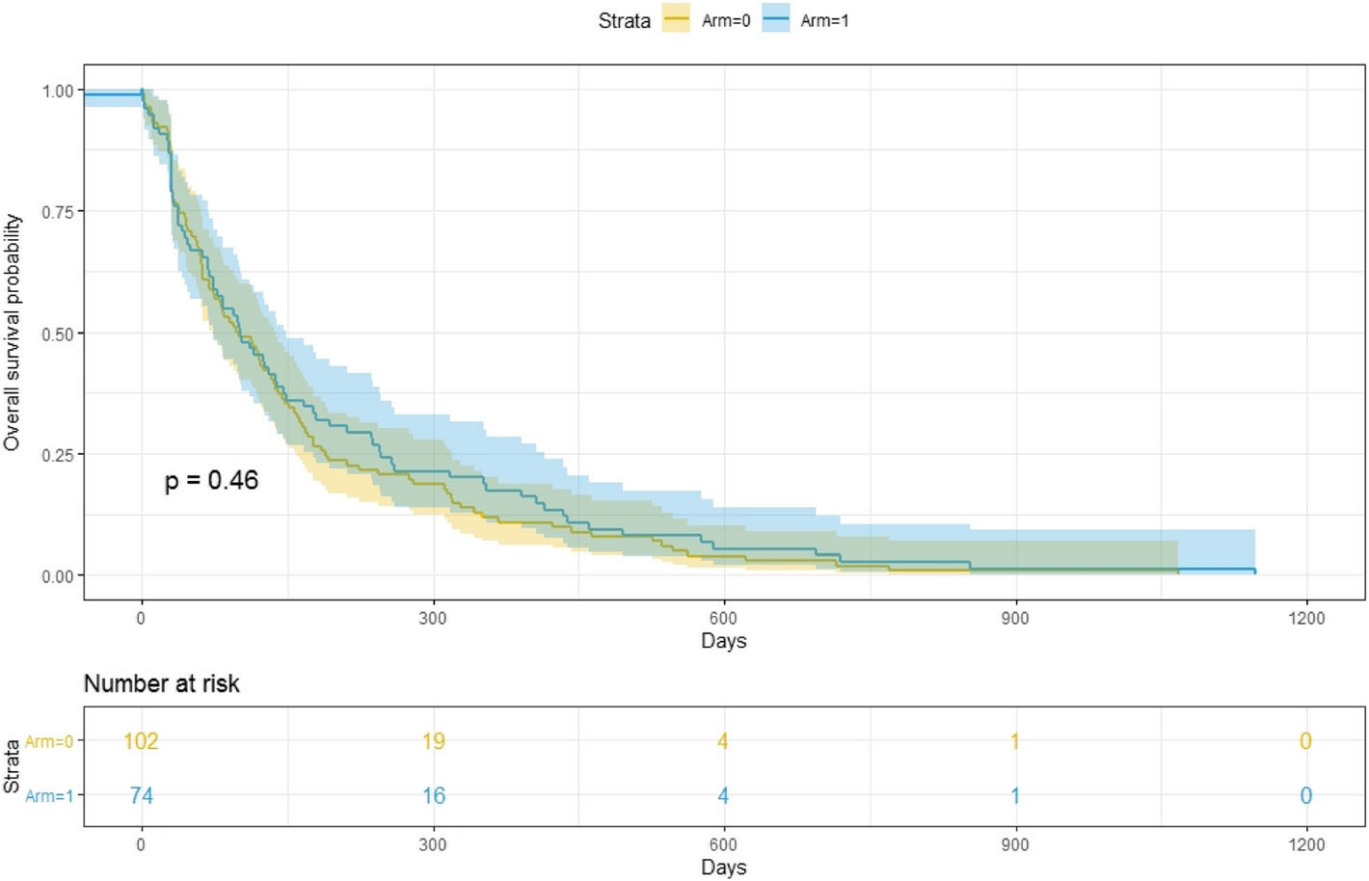
Progression-Free Survival

The three covariates considered for the modelling were Arm, Age, and Gender. The results were illustrated in Table 1. The survival curves corresponding to LRC and PFS are shown in Fig. 1 and Fig. 2. The Kolmogorov-type supremum test was performed to obtain the *p*-value.

The AFT models computed considering arm as the only covariate. The model was

12 $$ Y=\log\ (G)=\mu +\beta \ast \mathrm{Arm}+\epsilon . $$

**Fig. 3:**
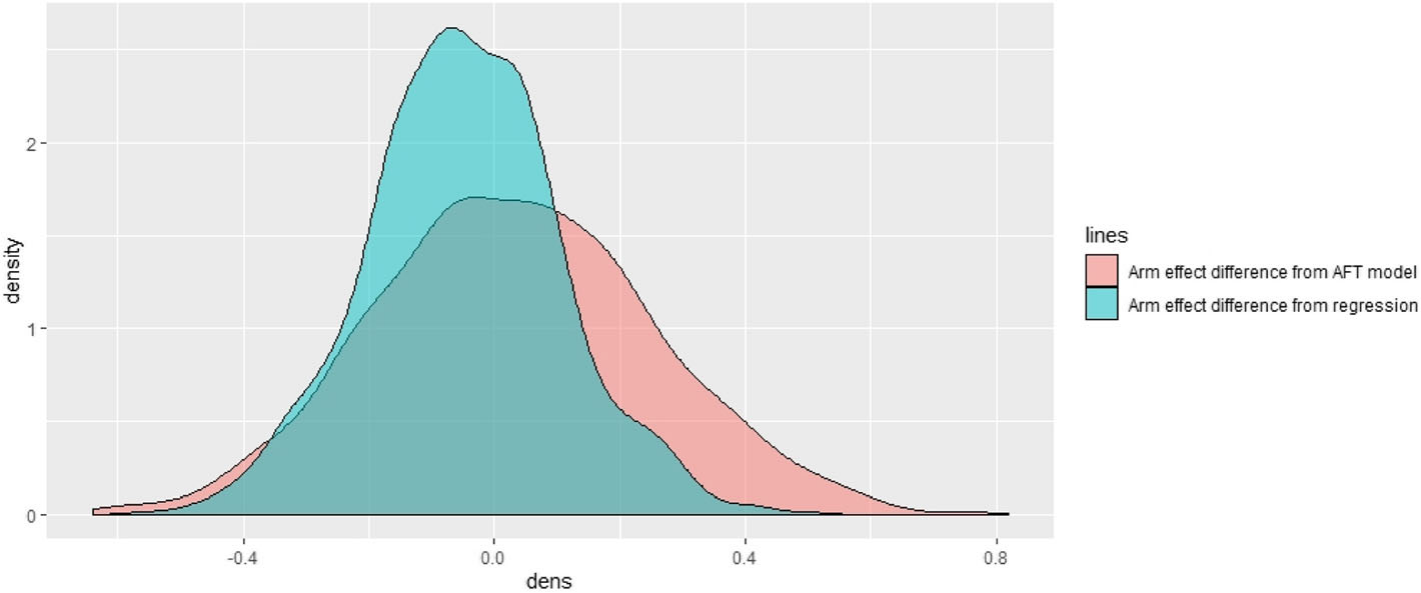
The plot of Arm effect difference from AFT model and auto-regression model

The posterior mean and standard deviation of the Arms were obtained by the AFT and regression model. The density plots of the difference of Arm effect from both the models are shown in Fig. 3.

We can draw this inference that the dependency of gap times is translated through the regression structure. So, adding the arm effect from the AFT survival model for the first gap time and the arm effect was obtained from the regression model. Thus, given the information of time between LRC and PFS, and the dependency structure between gap times, the survival duration between PFS and OS was predicted. The results of the posterior means obtained using the Bayesian AFT model are given in Table 2.

**Table 2:** Posterior Estimates generated for different gap times through AFT model

Response	Parameter	Posterior Mean (SD)	95% HPD
PFS	Intercept Arm	5.32 (0.109) 0.10 (0.15) 2.46 (0.41)	(5.10, 5.53) (−.18, 0.40) (1.71, 3.34)
OS	Intercept Arm	4.66 (0.121) 0.14 (0.17) 0.83 (0.09)	(4.42, 4.89) (−0.19, 0.47) (0.65, 1.03)

## Discussion

The novel coronavirus that causes COVID-19 appeared more than twice as high among individuals with cancer than the general population [17]. In survival analysis of disease-related to oncology, the patients commonly experience multiple events like loco-regional relapse, progression, death across the follow-up period. The interest lies in the prediction of survival duration for a particular event and evaluating effective treatments - the analysis carried by assuming the independence of the events. However, due to missing data on follow-up visits of the patients, information regarding the complete follow-ups of the patient is often unknown. So, their survival duration cannot be predicted based on the analysis carried out on the previously occurred events. The dependent modelling of the durations between consecutive events will assist in predicting the occurrence of the next event. The generalised version of the multi-state model is well-documented [18, 19]. The purest form of the mortality model having two states are, ‘alive without disease’ and ‘dead’ and a linked transition between these two states. The competing risk model is defined as a provision where individuals may die due to other causes [20–22]. The widely accepted form of the multi-state model is the illness-death model or disability model. The associated package to work in these directions is ‘mstate’ is useful for multi-state regression and to get prediction probability. Another package ‘survdim’ is helpful to perform type-specific Cox models. The parametric multi-state model showed through ‘msm’ and ‘flexsurv’. This work is performed with open source software OpenBugs to serve the Bayesian.

## Conclusions

The constant news about the coronavirus pandemic is relentless and has a long list of terrifying characteristics, and it is frightening because they are unknown and unpredictable. In this situation of the outbreak, it is not possible to separate treatment for cancer patients due to COVID-19. An effective treatment comparison strategy is required. We presented a handy tool to understand cancer progression in this unprecedented scenario. Linking different events of cancer progression is the need of the hour, and it is a methodological challenge. We provide the solutions to overcome the issue with intervals between two consecutive events by considering the example of head and neck cancer (HNC) data.

Now it is difficult to run a cancer clinical trial with COVID-19. All ongoing cancer clinical trials now are either on hold or severely affected. It is not a temporary problem. It will put questions about COVID-19 related death in all ongoing trials in the future. Unless we create a comprehensive analytical strategy to deal with COVDI-19 associated mortality during the cancer clinical trial, we cannot find the best effective treatment outcomes obtained through cancer trials. We preferred not to consider LRC, PFS, and OS as separate entities to understand treatment success. Here, LRC and PFS entities are merged through their gap times and defined as event till PFS. The recommendation is to consider disease progression and transition into account rather than consider these events as separate entities to understand the best treatment effect.

## Data Availability

Not applicable.

## Abbreviations

AFT: Accelerated failure time
AIC: Akaike information criterion
COVID-19: Coronavirus disease
DIC: Deviance information criteria
HNC: Head and neck cancer
HPD: Highest posterior density
LRC: Loco-regional control
MCMC: Markov chain Monte Carlo
OD: Overall death
PFS: Progression-free survival
SD: Standard deviation
